# Effects of radiation emitted by WCDMA mobile phones on electromagnetic hypersensitive subjects

**DOI:** 10.1186/1476-069X-11-69

**Published:** 2012-09-21

**Authors:** Min Kyung Kwon, Joon Yul Choi, Sung Kean Kim, Tae Keun Yoo, Deok Won Kim

**Affiliations:** 1 Brain Korea 21 Project for Medical Science, Yonsei University College of Medicine, Seoul, South Korea; 2 Department of Medical Engineering, Yonsei University College of Medicine, Seoul, South Korea; 3 Graduate Program in Biomedical Engineering, Yonsei University, Seoul, South Korea; 4 Department of Medicine, Yonsei University College of Medicine, Seoul, South Korea

**Keywords:** Provocation, Physiological changes, HRV, Subjective symptoms, EMF perception

## Abstract

**Background:**

With the use of the third generation (3 G) mobile phones on the rise, social concerns have arisen concerning the possible health effects of radio frequency-electromagnetic fields (RF-EMFs) emitted by wideband code division multiple access (WCDMA) mobile phones in humans. The number of people with self-reported electromagnetic hypersensitivity (EHS), who complain of various subjective symptoms such as headache, dizziness and fatigue, has also increased. However, the origins of EHS remain unclear.

**Methods:**

In this double-blind study, two volunteer groups of 17 EHS and 20 non-EHS subjects were simultaneously investigated for physiological changes (heart rate, heart rate variability, and respiration rate), eight subjective symptoms, and perception of RF-EMFs during real and sham exposure sessions. Experiments were conducted using a dummy phone containing a WCDMA module (average power, 24 dBm at 1950 MHz; specific absorption rate, 1.57 W/kg) within a headset placed on the head for 32 min.

**Results:**

WCDMA RF-EMFs generated no physiological changes or subjective symptoms in either group. There was no evidence that EHS subjects perceived RF-EMFs better than non-EHS subjects.

**Conclusions:**

Considering the analyzed physiological data, the subjective symptoms surveyed, and the percentages of those who believed they were being exposed, 32 min of RF radiation emitted by WCDMA mobile phones demonstrated no effects in either EHS or non-EHS subjects.

## Background

With the increasing use of third generation (3 G) mobile phones, social concerns have arisen concerning the possible health effects of radio frequency-electromagnetic fields (RF-EMFs) emitted by mobile phones in humans [1]. On the basis of limited evidence from both human and animal studies, the World Health Organization has classified RF-EMFs as possibly carcinogenic to humans (Group 2B) [2]. A number of people have self-reported electromagnetic hypersensitivity (EHS), characterized by a variety of non-specific symptoms that differ from individual to individual. Cross-sectional survey studies in different countries have reported that EHS subjects experience non-specific subjective symptoms (e.g., headache, dizziness, fatigue, sleep disorder) associated with EMF exposure: 1.5% in Sweden [3], 3.2% in California [4], and 5% in Switzerland [5]. For some individuals, the symptoms can have lifestyle-changing consequences [6].

Although numerous studies have examined the effects of Global System for Mobile Communications (GSM) on humans between EHS and non-EHS groups, only a few provocation studies involving WCDMA have simultaneously evaluated physiological changes, subjective symptoms, and EMF perception. Furubayashi et al. measured psychological and cognitive parameters during pre- and post-exposure [7]. They also monitored physiological parameters, such as skin temperature, heart rate and local blood flow, and asked participants (EHS and non-EHS women) to report on their subjective perception of EMF emitted by WCDMA devices. They concluded that EHS and non-EHS groups did not differ in their responses to real or sham EMF exposure with respect to any psychological, cognitive, or autonomic parameter.

Electromagnetic sensibility in the context of subjective symptoms and perception refers to the ability to perceive EMF without necessarily developing non-specific health symptoms attributable to EMF exposure [8]. Mueller et al. reported no significant differences in the ability to detect EMF between EHS and non-EHS groups [9]. In a study by Hietanen et al., in which EHS subjects were examined for their ability to perceive EMF, none of the subjects could distinguish real EMF exposure from sham exposure [10]. Kwon et al. reported that there was no evidence to indicate that EHS subjects could detect EMF exposure [11]. However, Leitgeb et al. reported that a subset of EHS subjects with significantly increased electromagnetic sensibility could be differentiated from non-EHS groups [8]. Therefore, a comprehensive study is necessary to understand whether EHS is actually caused by exposure to RF-EMFs.

## Methods

### Subjects

Because determination of EHS subjects was crucial to this provocation study [5], we utilized the EHS screening tool developed by Eltiti et al. [12]. We adopted the following criteria to identify EHS individuals: (1) a total symptom score greater than or equal to 26 out of a maximum score of 228 (57 symptoms, each ranked from 0 for “not at all” to 4 for “a great deal”); (2) individuals who explicitly attribute their symptoms to exposure to only 3 G mobile phones; and (3) individuals whose current symptoms cannot be explained by a pre-existing chronic illness.

The experiment was performed as a double-blind study with a total of 45 subjects. Initially, 19 EHS and 26 non-EHS subjects were screened; however, two EHS subjects and six non-EHS subjects were excluded. The two EHS subjects were excluded because they were considered outliers in respiration rate, which was greater than two standard deviations from the median (extreme outlier) or 20 beats per min higher than normal without exposure. In the non-EHS group, one subject was excluded because of some drowsiness and motion artifacts during the experiment; three subjects were excluded because they were outliers with respect to heart rate; and two subjects were eliminated because of abnormal electrocardiogram (ECG). None of the EHS or non-EHS subjects failed to attend the second day after attending the first day. Therefore, data from a total of 37 subjects—17 EHS and 20 non-EHS—were analyzed in this study. As shown in Table 1, there were no significant differences in male–female ratio, age, height, weight, body-mass index, nonsmoker-smoker ratio, computer usage time, TV viewing time, or mobile phone usage between the two groups.

**Table 1:** Demographic data of subjects

	**EHS**	**Non-EHS**	***P*-value**
No. of subjects (n)	17	20	-
Male: female	8: 9	11: 9	0.75
Age (yr)	30.1 ± 7.6	29.4 ± 5.2	0.87
Height (cm)	167.9 ± 7.5	167.6 ± 8.0	0.71
Weight (kg)	63.2 ± 11.9	60.3 ± 11.5	0.44
BMI (kg/m^2^)	22.3 ± 2.9	21.3 ± 2.3	0.24
Nonsmoker: smoker	15:2	18:2	1.00
Computer usage time (h/d)	4.4 ± 2.9	5.0 ± 3.8	0.99
TV viewing time (h/d)	1.6 ± 1.3	1.5 ± 1.1	0.96
Mobile phone usage periods (yr)	10.9 ± 3.0	11.6 ± 2.6	0.33

The subjects were advised not to consume caffeine, smoke or exercise, and to sleep enough before the experimental day in order to minimize confounding factors. All subjects, who were recruited by advertisements at the Yonsei University Hospital System (YUHS), were informed of the purpose and procedure of the experiment and were required to give written consent to participate in this study. The Institutional Review Board of the YUHS approved the protocol of this study (project number: 1-2010-0030).

### Experimental setup

The laboratory was used exclusively for this experiment, and all other electrical devices were unplugged except for our instruments in order to minimize background field levels. Background extremely low frequency (ELF) fields at the level of the head in the laboratory were measured to ensure that they did not influence the subjects. The average ELF electric and magnetic fields were determined to be 1.8 ± 0.0 V/m and 0.02 ± 0.01 μT, respectively, measured using an electric and magnetic field analyzer (EHP-50C, NARDA-STS, Milano, Italy). The RF field was determined to be 0.05 ± 0.00 V/m with a microwave frequency range from 1920 to 1980 MHz, measured using a radiation meter (SRM 3000, Narda GmbH, Pfullingen, Germany).

**Figure 1:**
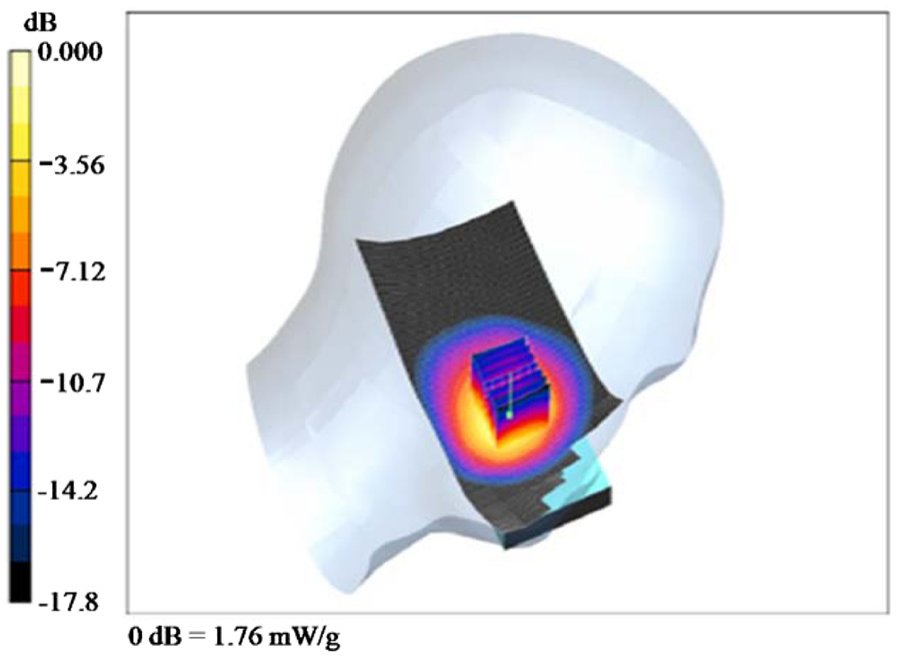
The measured SAR distribution of the WCDMA module on the left side.

To achieve better control over exposure, we used WCDMA modules with Qualcomm chipsets (baseband: MSM6290, RF: RFR6285, power management: PM6658, San Diego, CA) to generate WCDMA RF-EMFs instead of a regular smart phone. The WCDMA modules continuously transmitted at a mean output power of 24 dBm at 1950 MHz, which was measured using a wireless communication test set (E5515C, Agilent, Santa Clara, CA). The modules were inserted into a dummy phone [13], and the location of the module was varied to meet the recommended general public specific absorption rate (SAR)_1g_ of 1.6 W/kg according to the IEEE Standard [14]. The SAR measurements were made with a DASY 4 measurement system (SPEAG, Zurich, Switzerland), and a Twin SAM (specific anthropomorphic mannequin) phantom was filled with head tissue-equivalent liquid (mass density, 1000 kg/m^3^) as specified by the Federal Communications Commission. The measured dielectric properties of the liquid were σ = 1.41 S/m and ϵ_r_ = 39.7 for the WCDMA frequency range. When the antenna of the module was positioned 67.5 mm from the ear reference point (ERP) of the dummy, the averaged peak spatial SAR_1g_ was determined to be 1.57 W/kg at 1950 MHz at the left cheek position [15]. The electric field and power drift at the ERP were 6.9 V/m and −0.001 dB, respectively. The measured SAR distribution is shown in Figure 1.

**Figure 2:**
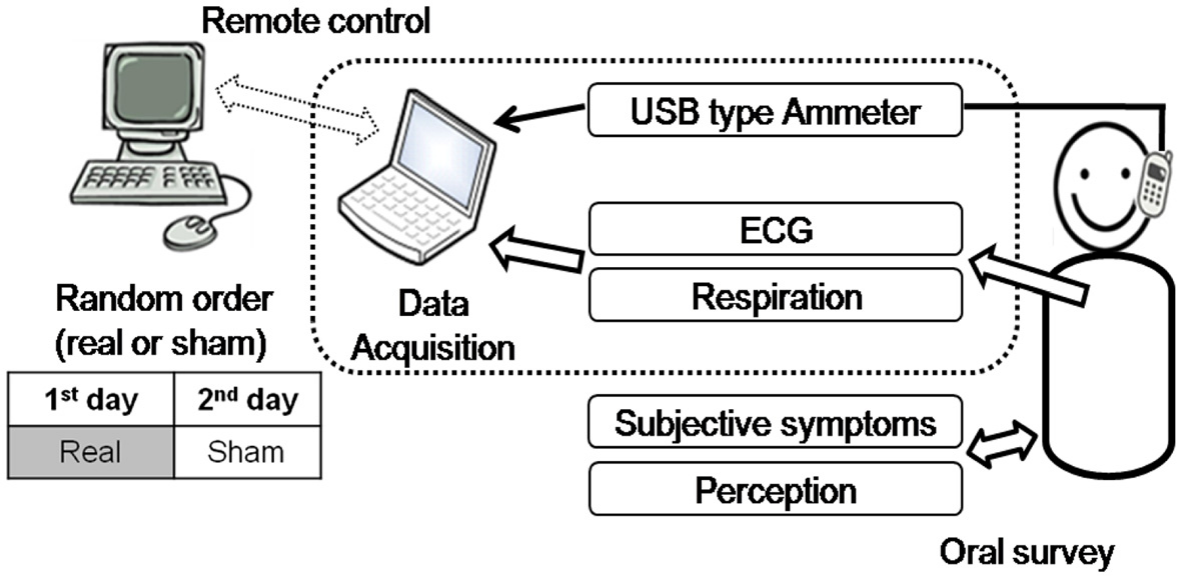
Block diagram of exposure setups.

The module was connected via a 5-m USB cable and a USB type ammeter to a portable laptop computer (X-note R500, LG Electronics, Korea), which controlled the module and monitored electrical current to check exposure conditions (Figure 2). The laptop computer was remotely controlled from another outside desktop computer to satisfy the double-blind study design. The dummy phone was attached to the subject’s head using an earplug and headset to fix it at the ERP next to the cheek [16]. The phone was held at a distance of 3 mm from the ear using a piece of wood for insulation to prevent battery-generated heat from providing subjects with an indication that the phone was working. The apparatus was constructed from plastic and rubber only, without any metal [16,17].

### Experimental procedures

No information was given to the subjects except that they would be asked about symptoms and RF-EMFs perception at the beginning of the first experimental day. Sham and real sessions were conducted as a double-blind test to minimize any test bias resulting from a subject and an experimenter recognizing the operational state of the WCDMA module. The experiment was performed for two days, one day for a real session and a second day for a sham session (or vice versa). No matter which came first, sham or real exposure, the second session was always conducted at approximately the same time of the day as the first session in order to maintain the subjects’ physiological rhythm. The order of sham and real sessions for each subject was randomly assigned and counterbalanced on our automatic exposure control program using MATLAB 2008a (Mathworks Inc. Natick, MA) to minimize experimental bias. Nine subjects in the EHS group and 11 in the non-EHS group received sham exposure session first. Time duration between sessions was a minimum of one day and a maximum of ten days.

Room temperature and relative humidity, which could considerably affect outcomes, were recorded and maintained. For the non-EHS group, room temperature showed no significant differences between real (24.4°C ± 0.9°C; Min = 23°C, Max = 26°C) and sham (24.5°C ± 0.8°C; Min = 23°C, Max = 26°C) sessions (*P =* 0.627). Humidity also showed no significant differences between real (40.0% ± 2.2%; Min = 35%, Max = 45%) and sham (40.8% ± 3.3%; Min = 35%, Max = 45%) sessions (*P =* 0.161). For the EHS group, room temperature showed no significant differences between real (24.1°C ± 0.9°C; Min = 23°C, Max = 26°C) and sham (24.2°C ± 1.1°C; Min = 23°C, Max = 27°C) sessions (*P =* 0.682). Humidity also showed no significant differences between real (40.0% ± 2.4%; Min = 32%, Max = 45%) and sham (39.7% ± 2.7%; Min = 36%, Max = 46%) sessions (*P =* 0.732).

### Physiological measurements

**Figure 3:**
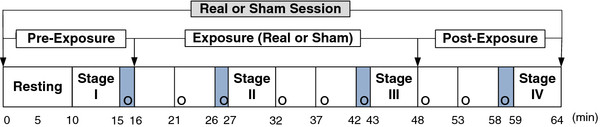
**Experimental procedures for measuring physiological changes and investigating symptoms and perception.** The four shaded areas are periods during which the subjects were questioned regarding the eight symptoms. “o” indicates timing of the inquiries for perception.

The duration of each exposure session was 64 min, as shown in Figure 3. Before the experiment, subjects were instructed to rest in a sitting position for at least 10 min. Physiological data were collected for 5 min each for four different stages: pre-exposure (stage I), after 11 min of exposure (stage II), after 27 min of exposure (stage III), and post-exposure (stage IV). At each stage, ECG and respiration were simultaneously measured for 5 min (the minimum data requirement for HRV) [18]. Heart rate, HRV, and respiration rate were obtained with a computerized polygraph (PolyG-I, Laxtha, Daejeon, Korea) with a sampling frequency of 512 Hz. The data were transferred to a nearby laptop computer (LG Electronics) and analyzed using data acquisition (Telescan 0.9) and analysis (Complexity software) software (Laxtha). The PolyG-I recorded ECG through Ag-AgCl electrodes (2223; 3 M, St. Paul, MN) placed on both arms and the right leg of participants.

Some studies have indicated that EHS subjects may exhibit abnormal autonomic nervous system regulation [19,20]. Therefore, we first obtained heart rate from ECGs and then acquired HRV and the power spectrum of HRV. High-frequency power (HFP) is reflective of the effects on respiratory sinus arrhythmia, an index of parasympathetic nerve activity, whereas low-frequency power (LFP) is reflective of the effects on both sympathetic and parasympathetic nerves [21]. In this study, the LFP/HFP ratio was used as an index of autonomic nerve activity balance. Respiratory inductance plethysmography, with an excitation frequency of 3 MHz, was used to measure respiration rate. Subjects wore a coiled band around their upper abdomen for measurement of inductance changes resulting from cross-sectional change.

### Subjective symptoms and perception of EMF

The four shaded areas in Figure 3 denote periods during which subjects were questioned regarding the eight symptoms; each period lasted approximately 1 min. The eight subjective symptoms of throbbing, itching, warmth, fatigue, headache, dizziness, nausea, and palpation were evaluated through verbal surveys, which were graded on a 4-point scale ranging from 1 (no sensation) to 4 (strong sensation) [22]. In addition, perception of EMF exposure was investigated every 5 min throughout the entire session, denoted by an “o” in Figure 3. Subjects were asked to answer the question “Do you believe that you are exposed right now?” nine times during each session. Percentages of those who believed they were being exposed were calculated for pre-exposure, exposure, and post-exposure periods. The total number of inquiries was 185 (5 × 37) during real exposure and 481 (13 × 37) during non-exposure; the total number of subjects was 37 (17 + 20).

### Data analysis

A repeated two-way analysis of variance (ANOVA) was performed using SPSS software (SPSS 18, SPSS, Chicago, IL) to investigate differences in heart rate, respiration rate, and relative change in LFP/HFP with exposure and stage for EHS and non-EHS groups. A *P*-value < 0.05 was considered statistically significant. Subjective symptoms, which are ordered paired data, were analyzed using a non-parametric Wilcoxon signed-rank test. A total of 64 *P*-values (4 stages × 8 symptoms × 2 groups) were obtained for the real and sham exposure sessions for the eight symptoms at four stages in both groups. The significance level was adjusted to 0.0125 (0.05/4) because testing was performed in four stages.

**Figure 4:**
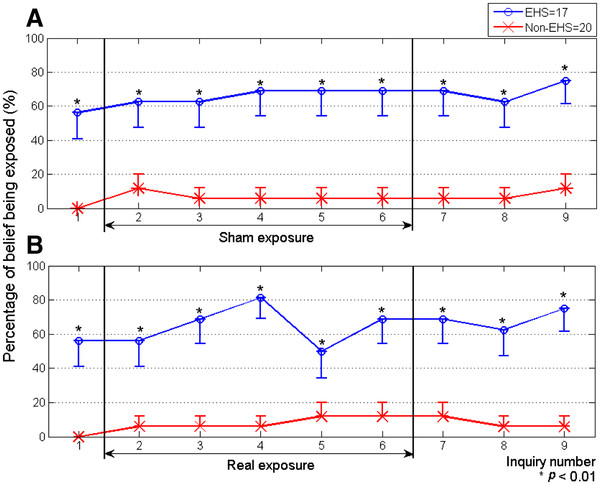
**Percentage of belief of being exposed in EHS and non-EHS groups for sham (A) and real (B) exposure sessions.** Asterisks indicate statistical significance in perception percentages between EHS and non-EHS groups. Bars indicate standard errors.

There were two exposure sessions for each participant, and nine perception inquiries for each session, as shown in Figure 3. For each session, there was one inquiry during pre-exposure, five inquiries during sham or real exposure, and three inquiries during post-exposure. In both groups, the percentages of those who believed they were being exposed were obtained and evaluated for significant differences between real and sham sessions using the McNemar test. The pre-exposure period of the sham sessions was compared with that of the real sessions to test whether the conditions before sham and real exposures of subjects were the same. The sham exposure period was compared with the real exposure period to test whether the subjects could detect the fields. The post-exposure period after sham exposure was compared with the post-exposure period after real exposure to test whether the real exposure influenced the perception of exposure in the post-exposure period. The Chi-square test was applied to evaluate differences in the percentages of those who answered “yes”, which were ordinal data, as shown in Figure 4.

## Results

### EHS and non-EHS groups

The symptom scores for EHS and non-EHS groups obtained using the Eltiti scale were 53.9 ± 28.5 and 9.3 ± 7.4 (mean ± S.D), respectively, and they were significantly different (*P* < 0.001). The most typical symptoms reported in the EHS group (n = 17) among 57 subjective symptoms on the questionnaire (multiple answers allowed) were fatigue (n = 17), headaches (n = 17), heaviness in the head (n = 17), exhaustion (n = 15), migraine (n = 15), sleep disturbance (n = 15), vertigo (n = 14), and difficulty in focusing attention (n = 14). The most typical symptoms reported in the non- EHS group (n = 20) were fatigue (n = 14), blurry vision (n = 10), difficulty in concentration (n = 10), heaviness in the head (n = 9), difficulty in focusing attention (n = 8), headaches (n = 6), migraine (n = 6), and pain/warmth in the head (n = 6).

### Physiological variables

Heart rate, respiration rate, and LFP/HFP ratios of the non-EHS and EHS groups during real and sham exposure are shown in the top section of Table 2. For analysis of the relative changes in LFP/HFP, LFP/HFP values for real and sham were expressed relative to the corresponding stage I values (defined as 100%) because of large individual variation. A repeated two-way ANOVA showed no significant differences in heart rate, respiration rate, or LFP/HFP for stage and exposure in either group, except for LFP/HFP for stage in both groups, as shown in the bottom section of Table 2. For the non-EHS group, LFP/HFP showed no significant difference between real and sham exposures (*P =* 0.552), but did show a significant difference among stages (*P =* 0.001). For the EHS group, LFP/HFP was also not significantly different between real and sham exposures (*P =* 0.079), but was significantly different among stages (*P =* 0.048).

**Table 2:** Descriptive and statistical tests for heart rate, respiration rate, and LFP/HFP among stage, exposure, and interaction

	**Heart rate (bpm)**				**Respiration rate (bpm)**				**LFP/HFP (%)**			
	**Non-EHS**		**EHS**		**Non-EHS**		**EHS**		**Non-EHS**		**EHS**	
	**Real**	**Sham**	**Real**	**Sham**	**Real**	**Sham**	**Real**	**Sham**	**Real**	**Sham**	**Real**	**Sham**
Stage: mean (standard error)												
I	76.0 (1.7)	75.6 (2.5)	77.0 (2.8)	77.2 (2.8)	17.2 (0.6)	17.3 (0.6)	17.4 (0.6)	18.0 (0.8)	100.0 (0.0)	100.0 (0.0)	100.0 (0.0)	100.0 (0.0)
II	75.5 (1.6)	75.3 (2.6)	77.8 (2.9)	77.2 (2.8)	17.3 (0.7)	17.9 (0.5)	17.6 (0.6)	17.0 (0.7)	143.9 (27.0)	165.6 (12.8)	133.8 (15.0)	122.7 (17.0)
III	75.2 (1.7)	74.4 (2.2)	76.4 (2.7)	77.6 (2.9)	16.9 (0.7)	17.6 (0.5)	17.5 (0.6)	17.3 (0.6)	151.0 (31.5)	167.6 (23.4)	198.3 (32.8)	110.6 (13.7)
IV	75.1 (1.6)	73.3 (2.1)	76.9 (2.8)	77.6 (2.9)	18.4 (0.7)	17.7 (0.5)	17.1 (0.7)	17.5 (0.7)	131.3 (23.5)	178.0 (19.9)	178.5 (31.0)	141.5 (23.5)
Factor (*P*-value)												
Exposure	0.629		0.815		0.772		0.754		0.552		0.079	
Stage	0.166		0.727		0.205		0.614		0.001*		0.048*	
Interaction (exposure & stage	0.621		0.226		0.518		0.431		0.428		0.055	

* *P* < 0.05, bpm; beats per min.

### Subjective symptoms

Neither the EHS nor the non-EHS group showed significant differences in any of the eight subjective symptoms surveyed (throbbing, itching, warmth, fatigue, headache, dizziness, nausea, and palpitation) between sham and real sessions in any of the four stages.

### Perception percentages

Table 3 shows the percentage of subjects who believed they were being exposed during pre-exposure, exposure (real or sham), and post-exposure in the EHS and non-EHS groups. To compare the percentages of those perceiving exposure during experimental sessions, we applied the McNemar test and found no significant difference between real and sham exposures in the EHS (*P =* 0.572) or non-EHS (*P =* 0.375) groups. To test whether there were any delayed effects of real exposure on post-exposure perception, we applied the same test and found no significant difference in the percentages of those who believed they were being exposed following real and sham exposures in the EHS (*P =* 1.000) or non-EHS (*P =* 1.000) groups. There was also no significant difference during pre-exposure between real and sham exposures in EHS (*P =* 1.000) and non-EHS (*P =* 1.000) groups, indicating that the conditions experienced by subjects before real and sham exposures were the same. Similarly, Kruskal-Wallis tests showed that the percentages of those who believed they were being exposed among pre-, sham exposure, and post-exposure were not significantly different in the EHS (*P =* 0.263) or non-EHS (*P =* 0.426) groups, demonstrating that conditions were the same for subjects throughout sham-exposure sessions.

**Table 3:** **Percentages of those who believed they were being exposed during pre-exposure, exposure and post-exposure periods, and*P*-values for sham and real exposures in EHS and non-EHS groups**

**Group**	**Session**	**Pre-exposure (%)**	***P*-value**	**Exposure (%)**	***P*-value**	**Post-exposure (%)**	***P*-value**
EHS (n = 17)	Real	47.1	1.000	65.9	0.572	62.8	1.000
		Sham	41.2		61.2		62.8
Non-EHS (n = 20)	Real	0.0	1.000	5.0	0.375	6.7	1.000
	Sham	0.0		8.0		6.7	

Figure 4 shows the percentages of subjects in the EHS and non-EHS groups for each inquiry number who believed they were being exposed in sham (Figure 4A) and real (Figure 4B) exposure sessions. Although there were significant differences between EHS and non-EHS groups during the real exposure period in Figure 4B, there were also significant differences during the sham exposure period (Figure 4A), suggesting that the significant differences between EHS and non-EHS groups during the real exposure period were not actually caused by exposure. The same reasoning applies to the significant differences during pre- and post-exposure in both sham and real exposure sessions. These higher percentages in the EHS group during both the sham and real sessions probably resulted from a bias of EHS individuals, who believe they can feel EMF, as described in our previous reports [23,24]. Therefore, there is no evidence that individuals in the EHS group perceived the radiation emitted by WCDMA mobile phones better than those in the non-EHS group.

## Discussion

Neither the EHS nor the non-EHS group showed significant differences in heart or respiration rate between real and sham exposures or among stages. In the case of LFP/HFP, however, there were significant differences between some stages during both real and sham exposure sessions in both groups. One disadvantage of the LFP/HFP analysis is that it is considerably influenced by stress, which can increase or decrease LFP/HFP [25]. Hjortskov et al. reported that psychological stress could result in increased LFP/HFP [26]. Nam et al. reported that LFP/HFP monotonically increased at each exposure stage in both EHS and non-EHS groups during 30 min of sham exposure [23]. In a subsequent study, Nam et al. also confirmed that LFP/HFP significantly increased over time in the absence of exposure, an effect the authors attributed to acute sleep deprivation resulting from awakening subjects with a noise when they exhibited drowsiness [27]. An additional potential source of stress was the requirement that subjects not move during a 64 min experiment. In fact, the “no-movement” requirement was the factor that drew the most complaints by subjects.

In the current study, neither the EHS nor non-EHS group showed significant differences in any of the four stages between real and sham sessions for any of the eight symptoms surveyed. Wilén et al., reported that exposure to RF-EMFs cannot explain perceived mobile phone attributed symptoms in EHS or non-EHS subjects [28]. Koivisto et al. also reported that RF exposure did not produce any consistent subjective symptoms or sensations such as headache, dizziness, and fatigue in non-EHS subjects [22]. Therefore, most likely, subjective symptoms resulted from a nocebo effect, meaning adverse symptoms occurred due to negative expectations [29].

There were no significant differences in the percentages of perception in either group who believed they were being exposed during pre- or post-exposure periods between real and sham exposures. There were also no significant differences in the perception percentages for either the EHS or non-EHS group during the sham exposure session (pre-exposure, sham exposure, post-exposure). Therefore, our experimental protocol seems minimally biased since we confirmed that there were no delayed effects, no differences in pre-exposure condition, and no difference in the percentage of those who believed they were being exposed among the pre-exposure, sham exposure, and post-exposure periods. With regard to the outliers, we included subjects who were outliers in the analyses and tested again to see whether their inclusion actually changed statistical tests for the physiological variables, symptoms, and perception. These results including the outliers were not significantly different from those excluding the outliers.

We used the EHS screening tool developed by Eltiti et al. to identify individuals who were sensitivity to RF-EMFs [12]. There is no objective diagnostic criterion for classifying someone as EHS at present. In the future, the statistical weighing of people’s self-reported hypersensitivity should substantiate their EHS claim.

## Conclusions

In both the EHS and non-EHS groups, there were no significant differences in heart rate, respiration rate, or LFP/HFP between sham and real exposure to a WCDMA module (average power, 24 dBm at 1950 MHz; specific absorption rate, 1.57 W/kg) attached inside a dummy phone for 32 min. There was no association between eight subjective symptoms and RF-EMFs exposure in either group. There was also no indication that EHS subjects could detect exposure. Therefore, considering the physiological data analyzed, the subjective symptoms surveyed, and the percentages of those who believed they were being exposed, no effects were observed in EHS or non-EHS subjects as a result from 32 min of RF radiation emitted by WCDMA mobile phones.

## Abbreviations

ANOVA: Analysis of variance; ECG: Electrocardiogram; EHS: Electromagnetic hypersensitivity; ELF: Extremely low frequency; ERP: Ear reference point; GSM: Global System for Mobile Communications; HFP: High-frequency power; HRV: Heart rate variability; h/d: Hour per day; IEEE: Institute of Electrical and Electronics Engineers; LFP: Low-frequency power; Max: Maximum; Min: Minimum; n: Number; RF-EMFs: Radio frequency-electromagnetic fields; SAR: Specific absorption rate; SD: Standard deviation; WCDMA: Wideband code division multiple access; yr: Year; YUHS: Yonsei University Hospital System; 3G: Third generation.

## Competing interests

The authors declare that they have no competing interests.

## Authors’ contributions

MKK recruited subjects, collected experimental data, and performed statistical analyses. JYC and SKK collected experimental data. TKY analyzed experimental data. DWK contributed to the development of the study protocol and editing of the manuscript. All authors read and approved the final manuscript.
